# Affective Learning and Psychophysiological Reactivity in Dementia Patients

**DOI:** 10.1155/2012/672927

**Published:** 2012-03-12

**Authors:** Andreas Blessing, Andreas Keil, L. Forest Gruss, Jacqueline Zöllig, Gerhard Dammann, Mike Martin

**Affiliations:** ^1^Psychiatric Clinic of Münsterlingen, P.O. Box 154, 8596 Münsterlingen, Switzerland; ^2^Department of Psychology, University of Zurich, Gerontopsychology Binzmuehlestrasse 14/24, 8050 Zurich, Switzerland; ^3^Department of Psychology and NIMH Center for the Study of Emotion and Attention, University of Florida, P.O. Box 112766, Gainesville, FL 32611, USA

## Abstract

We examined the association of faces with biographical information that varied in emotional content in patients with Alzheimer's disease and a healthy control group. During two experimental sessions, participants rated neutral male faces on dimensions of hedonic valence and emotional arousal, later paired with fictitious biographical information. Both groups changed their ratings of the faces according to the biographical content. Free recall and recognition were tested in the second session. Patients neither recalled the biographical information nor recognized the faces, whereas the controls did. In addition, psychophysiological measures were taken in response to the face stimuli. Patients showed significant heart rate modulation as a function of their emotion ratings, whereas the controls did not. No correlation of rating changes with skin conductance was found in any group. Results suggest that psychophysiological reactions such as heart rate changes may indicate preserved affective associative learning in dementia patients despite impaired explicit memory.

## 1. Introduction

The detailed assessment of cognitive deficits and resources is well established in the diagnosis of Alzheimer's disease (AD). In addition, the assessment of emotion processing in dementia patients is becoming increasingly important, as emotional resources may be relevant for the quality of life of patients and caregivers [1]. Existing studies investigating emotional processing in dementia patients have either focused on the interaction of explicit memory and emotion [2–6] or on the recognition of facial expressions of emotions [7–11]. By contrast, little is known about implicit learning of affective dispositions. Few studies investigated if affective responses to a stimulus, including appetitive versus aversive response tendencies, can be manipulated in dementia patients. Such information is useful in the context of therapeutic efforts complementing traditional medication-based interventions by means of behavioral strategies.

A classical approach to assess implicit affective learning processes is classical differential fear conditioning. Hamann, Monarch, and Goldstein [12] employed this paradigm in patients with AD. They examined fear conditioning in a group of 10 patients with AD and 14 controls by measuring skin conductance and electrodermal activity. A green rectangle was used as the conditioned stimulus (CS+) and was paired with a loud noise (US) using a partial reinforcement schedule. Results revealed that AD patients showed a similar electrodermal response as the control group to the CS+ and a CS− (red rectangle) that was never paired with the US. In contrast, skin conductance responses of AD patients did not differentiate between CS+ and CS− while this differentiation was present in the control group. In response to the US, however, AD patients showed the same electrodermal response as the control group. The results of this study suggest that fear conditioning and, more generally, implicit emotional learning processes might be partially impaired in AD patients. This finding was recently replicated by Hoefer and colleagues [13].

Complementing findings on fear conditioning, Blessing et al. [14] demonstrated in an emotional learning paradigm that dementia patients' affective evaluation of a stimulus may be altered by novel information and that these changes can still be detected after 22 hours. In this study, neutral male faces were paired with fictitious biographical content that characterized the depicted persons in terms of either positive or negative traits. Faces were rated before and at two different time points after the presentation of fictitious biographical content with respect to valence and arousal. Recognition of faces and free recall of fictitious biographical content were tested. Patients changed their ratings of pictures according to the biographical information presented, but did not recognize faces above chance level, or recall biographical information. These findings were replicated in a subsequent study [15]. Taken together, results suggest that learning of affective responses can be achieved in dementia patients when pairing faces with emotional content.

The results of studies using paradigms in which faces are associated with affective content seem to contradict the results of fear conditioning studies in terms of implicit learning processes. This is particularly surprising given that the processes investigated in the two approaches rely on partly overlapping neural structures. The neural basis of fear conditioning has been extensively studied in animals and humans. Frontal and temporal regions, including the amygdala, insula, and ventromedial prefrontal cortex, are involved in successful fear conditioning, diencephalic, and brain stem structures further mediate psychophysiological and behavioral responses [16, 17]. The amygdala is a core structure in fear conditioning [17–19].

The neural basis in paradigms associating visual stimuli (such as faces) with affective content has been studied less extensively. However, studies indicate that similar structures are relevant as in fear conditioning [20, 21]. For instance, Todorov and colleagues showed [20] that emotional information based on memories from short descriptions of persons is spontaneously retrieved upon face perception in healthy participants. However, the medial temporal lobe memory system does not seem to be critical for learning of affective trait associations with faces. In fact, a patient with a hippocampal lesion showed similar learning effects as controls in contrast to two patients with amygdala lesions [21]. Hence, the amygdala seems to be a critical structure in both learning affective trait associations with faces as well as in fear conditioning.

An explanation for the conflicting findings of impaired fear conditioning in AD patients and intact emotional learning performance in face emotion association tasks could be the dissociation between parameters measured with the two methodological approaches [22]. Whereas face emotion association tasks often measure the emotional experience by self-report scales, fear conditioning studies have typically used psychophysiological responses. An exception is the study of Burton and Kaszniak [22], where they presented AD patients and a control group with pleasant, aversive, and neutral pictures. Participants rated the pictures on the dimensions of valence and arousal, and corrugator and zygomatic EMG muscle activity was recorded. They found that AD patients and the control group indicated similar emotion ratings for the stimuli. However, facial muscle activity patterns in response to these emotional stimuli differed between groups: AD patients demonstrated an inverted pattern of zygomatic activity compared to controls. The results of this study suggest that there might be dissociation between emotional experience and psychophysiological responses in AD patients.

In young adults, psychophysiological measures have been demonstrated to correspond with self-report of emotional experience assessed with rating scales (i.e., the self-assessment manikin (SAM) rating scale; [23]). However, if there is a dissociation between emotional experience and psychophysiological responses in AD patients, psychophysiological measures should not correspond with rating changes in self-report scales in a face emotion association paradigm. Alternatively, it is possible that interoceptive cues (such as autonomic responses to the visual stimuli) underlie the modulation of evaluative report in AD patients despite lack of conscious recollection. Thus, the question arises to what extent AD patients' psychophysiological reactions are corresponding to changes in their affective ratings and, hence, to the reactions measured in healthy subjects.

Based on results of previous studies investigating psychophysiological responses to affective stimuli [24], we predict an increase in heart rate and electrodermal reaction in response to the face stimuli when paired with affective content. This psychophysiological response should, however, only be present in participants with intact emotional processing capacity. Such an intact emotional processing capacity might be indicated through the ability to learn associations of pictures and affective content. Hence, in the face emotion association paradigm, stronger psychophysiological reactions can be predicted in participants who change their affective ratings in a way consistent with the emotional content presented than in participants who are unable to learn these associations. If AD patients change their evaluative dispositions through pairing faces with information and also show altered psychophysiological responses, then this would indicate that important aspects of affective processing are intact in AD.

The aim of our study was to assess the relationship between rating changes in the face emotion association paradigm and physiological measures such as phasic heart rate response and skin conductance in patients with AD and healthy controls. In the face emotion association paradigm, neutral male faces were paired with fictitious biographical content that characterized the depicted persons in terms of positive, negative, or relatively neutral traits. Faces were rated before and at two different time points after the presentation of fictitious biographical content with respect to valence and arousal. Recognition of faces and free recall of fictitious biographical content were tested. Heart rate and skin conductance in response to picture presentation were then measured. AD patients were predicted to change their affective ratings of faces after the presentation of the fictitious biographical content despite impaired recognition of faces and impaired recall of biographical content. It was hypothesized that the ratings of the control group were also influenced by the biographical information, but that they would recognize the pictures and recall some of the biographical information. We predicted correlations of psychophysiological measures and changes in affective ratings in both groups.

## 2. Design and Methods

### 2.1. Participants

The study included 16 patients, 14 diagnosed as AD, and 2 diagnosed as mixed dementia (mean age 76.1 years (SD = 5.8); mean education 10.3 years (SD = 3.1); mean minimental state ([25]; for a review see [26]) 21.8 points (SD = 4.0); 7 females and 9 males).

Patients were recruited in a regional facility at the time of testing. All patients were diagnosed by a multidisciplinary team of the hospital ward using ICD 10 criteria [27]. The diagnosis was based on general medical, neurological, and neuropsychological examinations. All patients had received medical testing including computerized tomography or magnetic resonance imaging and specific screening blood tests, in order to exclude syphilis, diabetes, thyroid disorders, and vitamin B12 and folic acid deficiency.

Twelve healthy elderly participants were recruited as controls (mean age 75.2 years (SD = 5.3); mean education 12.5 years (SD = 3.1); 7 females and 5 males). Mean age and mean education of patients and controls did not differ (*t*  (26) = −0.417; *P* > .68, resp., *t*  (26) = 1.851; *P* > .08). Controls were noninstitutionalized and managed their own household. They reported that they had no known CNS diseases, contact with toxic substances or substance abuse. All participants gave written informed consent. The study protocol was approved by the local ethics review board.

## 3. Material

### 3.1. Pictures

Test stimuli were three pictures of neutral male faces selected from the face database ([28]; TMW male 33-2 neutral, TMW male 34-2 neutral, TMW male 34 neutral). Three other pictures of neutral male faces served as control stimuli for the recognition task (TMW male 29 neutral, TMW male 32 neutral, TMW male 34-3 neutral). All stimuli were printed in US letter format on white paper. While psychophysiological measures were recorded, pictures were presented on a 17′ computer screen using Presentations Software (Version 9.70).

### 3.2. Emotional Ratings

The self-assessment manikin (SAM) is an affective rating system to assess participants' ratings of various stimuli [29]. Its dimensions of valence (ranging from pleasant to aversive) and arousal (ranging from low to high intensity) have shown reliable relationships with other measures of emotional responses such as physiological and behavioral parameters [23]. The SAM rating scale has been successfully used in previous studies with dementia patients [14, 15, 30]. Using the paper-pencil version of this instrument, participants rated the stimuli by hedonic valence and emotional arousal.

### 3.3. Peripheral Measures of Autonomic System Arousal

The electrocardiogram (ECG) was recorded from the left and right forearms using a BioPac system; R-waves were detected and Interbeat intervals were recorded to the nearest millisecond using AcqKnowledge Software, which is part of the BioPac system. The same system was used to record the skin conductance response: skin conductance electrodes were placed adjacently on the hypothenar eminence of the left palm, filled with 0.05 m NaCl Unibase paste. A Biopac MP100 acquisition unit equipped with a GSR 100B amplifier (0.5 V constant voltage, sensitivity 600pS (Biopac)) sampled electrodermal activity at 200 Hz continuously throughout the experiment. The occurrence of experimental events (e.g., stimulus presentation) was simultaneously recorded on a separate analog channel to permit analysis of stimulus-related heart rate (HR) changes and skin conductance responses (SCRs). The amplitude of the SCR was calculated in half-second bins and SCR change time series was drawn with respect to a 1-second prestimulus baseline (see [31], for a similar procedure). Skin conduction level and amplitude change provide indices of general activation in the sympathetic chain. Heart rate is dually innervated: decelerating with parasympathetic action and accelerating with activation of the sympathetic system.

### 3.4. Procedure

In the first session, each participant viewed the three experimental face stimuli and was asked to rate them in terms of pleasure (hedonic valence) and emotional arousal using the SAM rating scale [29]. Subsequently, fictitious biographical information about the men in the pictures was presented. While participants heard the biographical information the appropriate picture was placed before them again. To avoid effects of face preference, we used two different combinations of faces and biographies (one combination was used 7 times in the control group and 10 times in the AD group, the other combination was used 5 times and 6 times, resp.). Each combination was presented in two orders when pictures were rated and biographical content was presented (one order was used 7 times in the control group and 9 times in the AD group, the other order was used 5 and 7 times, resp.). The aversive fictitious information characterized the depicted person in terms of socially unacceptable behavior, whereas the pleasant fictitious information contained only socially acceptable behavior. The neutral fictitious information contained no descriptions that were socially unacceptable or remarkably positive. Biographical narratives were tested in previous studies and have been shown to elicit measurable affective dispositions [14, 15].

After a retention interval of 190 minutes, participants completed a forced choice recognition test at the start of the second session. They were presented with three pairs of faces each containing a previously shown target face and a novel distractor face. For each pair, participants were asked to indicate which face was more familiar to them. Furthermore, they were asked whether they had seen the picture before and were tested on recall of the fictitious biographical information. Subsequently, the faces that were presented during the first session were rated again on the dimensions of valence and arousal. After that, participants were prepared with sensors to record psychophysiological measures. Subjects were then seated about 50 cm from the computer monitor and instructed to look at the pictures that would appear on the monitor. To achieve satisfactory data quality and reduce artifacts, participants were asked to refrain from moving or talking. The three experimental pictures and a distractor face from the recognition test were presented on the computer screen in a sound-attenuated dimly lit room. Pictures were presented 20 times for four seconds each in a fixed random order. Interpicture interval varied randomly between 5.5 and 6.5 seconds.

### 3.5. Data Reduction and Artifact Control

R-waves were detected in the digitized ECG, and interbeat intervals were converted to beats per minute. Participants' mean HR responses and peaks of SCR were calculated for each of the four pictures. Epochs of 6000 ms (1000 ms before onset to 5000 ms after onset) were segmented and averaged. Only the first 4000 ms of heart rate change during stimulus presentation were used for statistical analysis (below), and they showed a slow heart rate acceleration across participants, followed by a return to baseline after about 6000 ms. Heart rate during the 1000 ms prestimulus baseline was subtracted from the heart rate response waveform after averaging for stimulus type and subject, to result in relative HR changes over time relative to baseline. A mean value was then calculated for the first 4000 ms for each condition and participant, and a log transformation was performed to normalize the distribution of the data for statistical analysis.

## 4. Data Analysis

**Free Recall**

To score the participants' report of the biographical information, recalled text as reported by the participants was transcribed. Using these transcripts, each correctly recalled element was assigned a point after the experimental session. Recalling information like “he stole a car” resulted in assignment of one point. Unspecific information like “he did something bad” was not scored.

**Pleasure and Arousal Ratings**

Repeated measures Analyses of variance (ANOVAs) were conducted for the dimensional ratings of pleasure and arousal. For each of these two dependent variables, two within-participant factors were used: (i) two different biographical contents (pleasant versus aversive characterization) and (ii) two measurement time points (immediate versus 190 minutes delay). Group was included as the between participant factor. Ratings of neutral pictures were not included in the analysis, because no rating changes after the presentation of fictitious biographical content were predicted. The neutral condition was included to compare correlations of affective ratings with psychophysiological measures in response to pictures paired with affective and neutral content. To assess correlations between the change in affective ratings and change in psychophysiological reactivity (below), rating data were transformed into change measures: For the valence and arousal ratings, the difference between pleasant-associated and aversive-associated faces was calculated for the second measurement time point. This difference was used as an index of association learning on affective ratings.

**Psychophysiological Data**

ECG and skin conductance data were analyzed separately using ANOVA as described above. In addition, correlation analyses were conducted in multiple steps. For both mean heart rate responses and the peaks of SCR, responses were combined across the four stimuli within each participant to form a composite score representing a measure of psychophysiological reactivity. Spearmans Rho was then used to assess correlations between rating changes and ECG/skin conductance. These analyses were conducted separately for each group. In case of a significant correlation of a difference score and a composite measure for psychophysiological reactivity, analysis was then broken down to compare correlations as a function of emotional content (pleasant, neutral, unpleasant, neutral). Rank-based measures and conservative *P* values were used in this last, explorative, step of data analysis.

## 5. Results

### 5.1. Recognition and Free Recall

In the forced choice recognition test for the three faces, participants of the control group performed near the optimum (mean correct choices = 2.92 (SD =  .29), *P* < .001) whereas the performance of AD patients did not differ from chance level (M = 1.63 (SD =  .81), *P* > .544).

In the free recall test, the healthy control group recalled 8.5 story elements (SD = 4.54) (min = 3, max = 16) after the delay, whereas none of the patients recalled any of the fictitious biographical information.

### 5.2. Ratings of Hedonic Valence

Mean valence ratings are presented in Table 1. As expected, the repeated measures ANOVA indicated a significant interaction between time and fictitious biographical content (*F *(2,25) = 17.5; *P* < .001), reflecting enhanced pleasure ratings for pleasant content and reduced pleasure ratings for aversive content over time. The interaction between group, time, and biographical content was not significant (*F *(2,25) = 1.749; *P* > .195). However, a significant interaction between biographical content and group was observed (*F *(2,25) = 4.327; *P* < .024) indicating a difference between patients and controls with respect to the impact of biographies on ratings. Posthoc ANOVA for the patients yielded no main effect of biographical content (*F *(2,14) = 3.262; *P* < .069), but a significant interaction between time and biographical content (*F *(2,14) = 4.988; *P* < .023) indicating that ratings of pictures associated with negative as well as positive biographical content changed in the expected direction after having been paired with the biographical content. ANOVA on controls' valence ratings showed a main effect of biographical content (*F *(2,10) = 11.210; *P* < .003) along with an interaction between time and biographical content (*F *(2,10) = 11.535; *P* < .003) suggesting that controls changed their valence ratings in line with the experimental manipulation.

### 5.3. Ratings of Emotional Arousal

The mean arousal ratings are presented in Table 2. ANOVA indicated a significant interaction between time and biographical content (*F *(2,25) = 4.397; *P* < .023), which reflected that all participants changed their ratings to endorse higher arousal for the faces paired with aversive, compared to pleasant and neutral biographies (posthoc *P* < 0.05). There was no interaction between group, time, and biographical content (*F *(2,25) = 0.071; *P* > .932) suggesting that both groups were influenced in the same way by the manipulation. No interaction between biographical content and group was observed (*F *(2,25) = 1.239; *P* > .307).

### 5.4. Heart Rate Response

**Figure 1:**
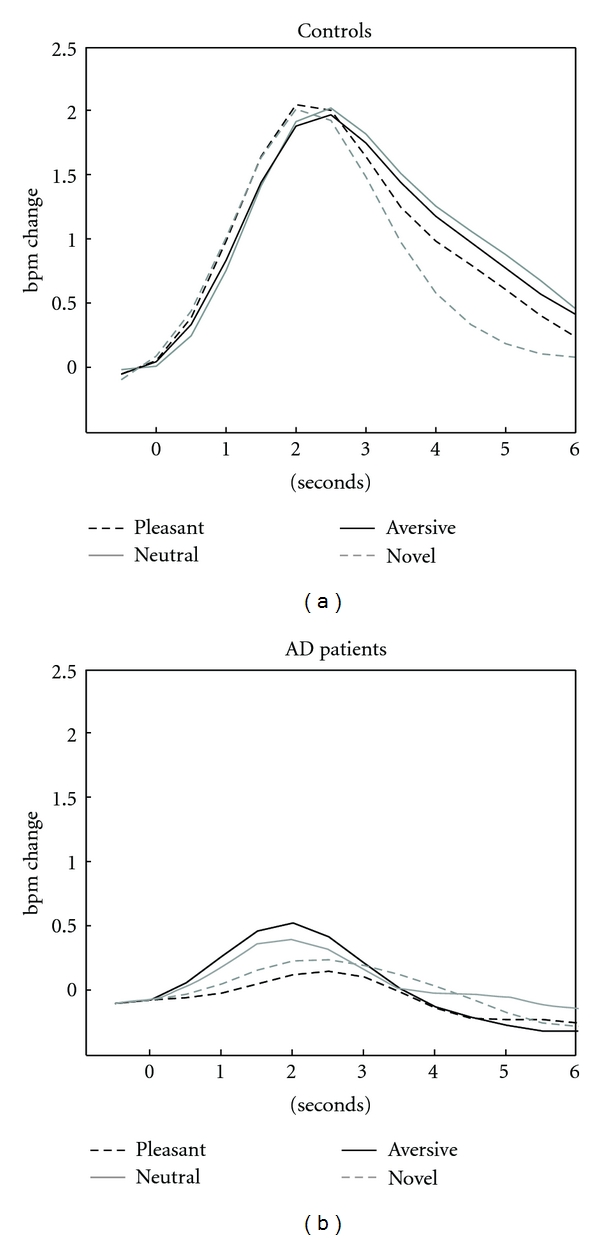
Mean heart rate response of patients and controls for pictures associated with pleasant, neutral, and aversive content and a novel stimulus.

**Figure 2:**
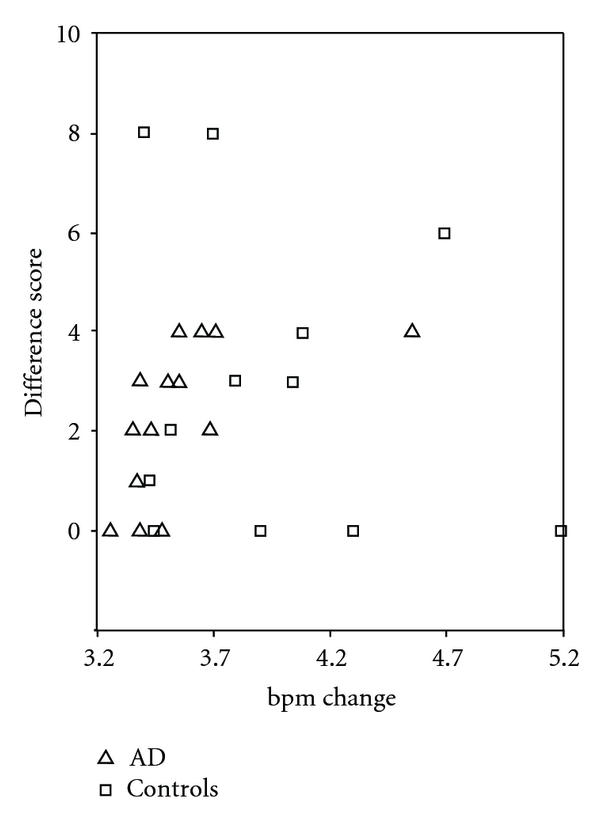
Interaction plot showing difference scores of arousal ratings in dependence of log function of mean heart rate response to faces associated with pleasant and unpleasant content for patients and controls.

Mean heart rate response of patients and controls for pictures associated with pleasant, neutral and aversive content and a novel stimulus are displayed in Figure 1. No correlation between mean heart rate response and difference score for arousal and valence ratings was found in the control group. In the patient group, the mean heart rate response composite score showed no association with the difference score for valence ratings, but a significant rankcorrelation occurred with the difference score for arousal ratings (*r* = .614, *P* < .011). This correlation shows that changes in arousal ratings due to the experimental manipulation were related to heart rate reactivity in the patient group. To analyze the contribution of different stimuli types to this relationship, correlations between difference scores for arousal ratings and heart rate responses to pictures associated with pleasant, neutral, and aversive content and the novel stimulus were calculated separately. For heart rate responses to the picture associated with neutral content as well as the novel stimulus, no relationship with difference score for arousal ratings was found. The correlations between HR change and the change score for arousal ratings reached trend levels for pleasant and aversive content (*r* = .475, *P* = .063, resp., *r* = .486, *P* = .056). When heart rate responses to pleasant and aversive stimuli were combined, the relationship with the difference in score was highly significant (*r* = .651, *P* < .006). This relationship is displayed in Figure 2 for both groups.

### 5.5. Skin Conductance Response

In both groups, skin reactivity showed no significant relationship with any change score for ratings (*r* < .39, *P* > .22).

## 6. Discussion

In the present study, neutral pictures of faces were paired with pleasant, aversive, and neutral biographical content and were presented to AD patients and controls. Replicating results of previous studies [14, 15], all participants changed their judgements of faces associated with pleasant and aversive content along the dimensions of valence and arousal according to the information presented. For instance, pictures paired with aversive content were rated higher in arousal and lower in valence than before presentation of the fictitious biographical content. Ratings of AD patients were influenced by the affective content that had been associated with the pictures although they did not recognize the pictures or recall the biographical information. The patients appeared to base their judgement on implicit dispositions, whereas participants in the control group could also use explicit memory to recall information about affective connotations.

The main aim of the present study was to address the question if psychophysiological measures correspond with rating changes in self-report scales due to affective trait face associations in dementia patients. Psychophysiological measures have been demonstrated to correspond with self-report of emotional experience assessed with rating scales in healthy young adults (i.e., the SAM rating scale; [23]). Thus, a positive relationship between affective report and psychophysiological reactivity in AD patients would indicate that important aspects of affective stimulus processing are preserved during emotional learning. In particular, such a finding would support the notion that the affective learning seen in AD reflects engagement of emotional response systems. Based on results of previous studies investigating psychophysiological responses to affective stimuli [24], we predicted a small increase in heart rate in response to face stimuli, which tend to be weaker elicitors of emotive engagement, compared to conditioned stimuli or pictures with emotional scenes. Healthy young adults presented with pictures of emotional events, consistently show heart rate deceleration as the rated unpleasantness of pictures increases and acceleration as rated pleasure increases [32]. Furthermore, decelerated heart rate responses in healthy participants during encoding of unpleasant pictures are uniformly reported [32–34]. In a study investigating electrodermal activity, findings revealed that young participants displayed larger electrodermal reactions when viewing unpleasant or pleasant pictures compared to neutral pictures [35]. The methodological difficulty when assessing psychophysiological reactions in response to specific pictures is that stimuli have to be presented repeatedly to get reliable results and that repetition prompts habituation. Bradley, Lang, and Cuthbert [24] investigated habituation patterns of startle reflex, heart rate, electrodermal, and facial corrugator muscle activity in response to repeated presentation of pleasant, unpleasant, and neutral picture stimuli. Only startle reflex differentiated among affective picture contents over trials, whereas for example the initially present deceleration of heart rate for arousing stimuli disappeared over trials. After repeated trials, subjects showed an acceleration of heart rate in response to all pictures presented.

Consistent with these results healthy control participants in our study consistently showed increases of heart rate in response to all stimuli presented. The increase of heart rate response was smaller in AD patients, suggesting a generally impaired psychophysiological reaction toward face stimuli.

It was further hypothesized that the psychophysiological response is related to the ability to learn associations of pictures and affective content. In fact, in our study rating changes on the arousal scale correlated significantly with mean heart rate response, but only in the patient group and not in controls. This correlation in the patient group shows that changes in arousal ratings due to the experimental manipulation are related to heart rate reactivity. Further analysis yielded that rating changes on the dimension of arousal correspond mainly with heart rate responses to pictures that have been associated with pleasant and aversive fictitious biographical content. Accordingly, patients who were influenced more by the experimental manipulation and changed their ratings of face stimuli on the dimension of experienced arousal showed a higher heart rate response to these stimuli.

The fact that we found no correlation of changes in valence ratings and psychophysiological measures might be explained with the higher sensitivity of the valence dimension to habituation effects. Accordingly, Bradley, Lang, and Cuthbert [24] could show in their study that specific aspects of psychophysiological reactions do differ between stimuli in relation to their different valence, however, this difference disappeared with repeated presentation. Furthermore, the arousal self-rating scale seems to measure intensity of affective reactions and this could further enhance correlations with psychophysiological reactions.

One question that remains is why there was no correlation between affective rating changes and psychophysiological measures in the control group. Our data show that all subjects in the control group had pronounced general psychophysiological reactions in response to all presented face stimuli, but these did not vary across emotional categories/biographies. This is not an unusual result, as faces are known to have a weak impact on peripheral reactions [31]. Controls who are aware of the stimulus repetitions and are able to assign biographies to the faces by means of recollection may thus not engage emotionally when perceiving the face stimuli, leading to blunted psychophysiological reactions as seen in healthy controls in general. Generally, the heart rate response of patients was weaker than that of controls, possibly because physiological reactivity is impaired in some patients. The reduced modulation of this response may represent an indicator of poor associative affective learning as indicated by the correlation with the arousal difference score. The correlation between the ability to learn affective face trait associations and heart rate response suggests that patients who are able to learn affective face trait associations show normal heart rate responses in contrast to patients who do not learn affective face trait associations. Thus, heart rate response and associative affective learning are both measures of emotional processing capacities in dementia patients. The finding that heart rate response is related to associative affective learning in dementia patients is particularly striking given the relatively modest affective intensity that can be expected to be evoked by face stimuli that have been associated with affective descriptions. Since patients do not consciously remember affective connotations they must rely on implicit memory systems. Patients may use their own psychophysiological reactions as a cue to evaluate face stimuli on affective dimensions.

We conclude that there is no dissociation between emotional experience and phasic heart rate response in dementia patients. This seems to contrast previous findings, as discussed above, Burton and Kaszniak [22] found that AD patients and a control group rated their emotional experiences towards the pleasant, aversive, and neutral pictures similar on the dimensions of valence and arousal. However, muscle activity patterns in response to these emotional stimuli differed between groups. AD subjects demonstrated an inverted pattern of zygomatic activity compared to controls. The results of this study suggest that there might be a dissociation between emotional experience and psychophysiological response in AD patients. Probably the results concerning the consistency between emotional experience and psychophysiological response differ between studies simply because different measures were assessed. The neural basis of heart rate response is not identical to the neural networks involved in muscle activity patterns in response to affective stimuli. Because neural structures are not uniformly affected by the pathological alterations of AD some psychophysiological responses can be impaired while others remain intact.

In contrast to the phasic heart rate response, which normally varies with emotional valence of pictures, electrodermal responses are primarily sensitive to the rated arousal of pictures and are augmented for pleasant and unpleasant stimuli as intensity level increases [32]. In the present study we found no correlation between changes in arousal or valence ratings and skin conductance. This seems to be consistent with the negative findings of fear conditioning studies in dementia patients that used skin conductance as a dependent measure [12, 13]. It seems, however, unlikely that dementia patients show normal skin conductance response to emotional stimuli but do not show normal skin conductance response when emotional reactions have been acquired. Both groups in our study showed no association of influence of affective content on picture ratings and skin conductance response. This suggests that skin conductance failed to differentiate in response to stimuli that have been associated with pleasant, unpleasant, neutral and novel material in our experiment. As for heart rate response similar habituation effects can be expected for skin conductance response. Bradley, Lang, and Cuthbert [24] found that skin conductance differentiated between arousing and nonarousing picture contents but that this effect disappeared over trials, that is, with increasing repetition. For the reasons discussed above, no conclusions can be drawn from the results of the present study concerning acquisition of skin conductance response in dementia patients.

In summary, the results of our study suggest that affective learning is possible in dementia patients despite impaired explicit memory. In the absence of conscious recollection, intact psychophysiological reactions such as heart rate changes suggest intact affective engagement in dementia patients, possibly providing interoceptive cues that underlie the modulation of evaluative report. The finding of preserved capacity to acquire new emotional dispositions in dementia patients despite severe loss of explicit memory has implications for therapeutic interventions and nursing strategies. For example, affective reactions of dementia patients in response to caregivers can be influenced through simple conditioning procedures as demonstrated in the present study: preferred music, foods, pictures, and so forth may be systematically associated with caregivers, in order to reduce inappropriate behavior. In addition, interventions are conceivable that associate spatial locations or objects with appetitive or aversive valence to aid the regulation of desired behaviors, such as avoiding potentially dangerous objects or locations.

## Figures and Tables

**Table 1:** Mean valence ratings of AD patients and controls for pictures paired with pleasant, aversive, and neutral content at two measurement time points.

Time			
Biographical content	Group	Baseline mean (SD)	190 min delay mean (SD)

Pleasant	AD	5.25 (2.41)	6.62 (2.0)
	Controls	5.50 (1.51)	6.75 (1.49)
Neutral	AD	6.19 (2.43)	7.25 (2.05)
	Controls	5.92 (1.68)	6.33 (0.99)
Aversive	AD	5.56 (2.07)	5.12 (1.71)
	Controls	4.92 (1.24)	2.58 (1.88)

Higher ratings denote higher pleasure. SD: standard deviation.

**Table 2:** Mean arousal ratings of AD patients and controls for pictures paired with pleasant, aversive, and neutral content at two measurement time points.

Time			
Biographical content	Group	Baseline mean (SD)	190 min delay mean (SD)

Pleasant	AD	2.94 (2.21)	2.87 (1.96)
	Controls	3.33 (1.01)	2.42 (1.51)
Neutral	AD	2.56 (1.97)	2.94 (1.44)
	Controls	3.17 (1.99)	2.99 (1.24)
Aversive	AD	2.87 (1.86)	4.87 (1.67)
	Controls	4.42 (1.83)	5.33 (2.50)

Higher ratings denote higher arousal. SD: standard deviation.
